# Patient satisfaction and pre-operative anxiety in day-care laparoscopic surgery: Association with recovery outcomes

**DOI:** 10.6026/973206300221815

**Published:** 2026-03-31

**Authors:** Jaydeep Dinesh Nagar, Midha Mehmood Shaikh, Arti Kasulkar, Mukul Singh, Gourav Singh Shekhawat

**Affiliations:** 1 Deaprtment of General Surgery, Shalby Multi-Specialty Hospitals, Ahmedabad, Gujarat, India; 2 Department of Obstetrics and Gynaecology, Bharati Vidyapeeth Medical College, Pune, India; 3 Department of Forensic NKP Salve Institute of Medical sciences & RC and LMH, Nagpur, India; 4 Department of General Surgery, All India Institute of Medical Sciences (AIIMS), Gorakhpur, Uttar Pradesh, India; 5 Department of Rajasthan University of Health Science, Jai Durga College of Nursing, Rajasthan, India

**Keywords:** Day-care surgery, laparoscopic, pre-operative anxiety, recovery outcomes

## Abstract

Day-care laparoscopic surgery prioritizes efficiency and safety, yet psychological determinants of recovery remain insufficiently integrated into outcome assessment. Therefore, it is of interest to evaluate pre-operative anxiety, post-operative satisfaction and objective recovery metrics in 130 adults undergoing elective ambulatory laparoscopic procedures. Higher pre-operative anxiety was significantly associated with delayed ambulation, increased post-operative pain and prolonged discharge readiness (p < 0.05), whereas higher satisfaction correlated with shorter discharge times (r = -0.34, p = 0.001). In multivariable analysis, both anxiety (β = +0.22, p = 0.002) and satisfaction (β = -0.29, p = 0.002) independently predicted discharge readiness after adjustment for age, sex and procedure duration. Thus, we show that psychological preparedness and patient satisfaction are independent determinants of ambulatory surgical recovery and should be incorporated into perioperative optimization pathways.

## Background:

The use of laparoscopic surgery performed in day-care facilities has increased in popularity because of increased use of minimally invasive surgical techniques enhanced recovery protocols and cost containment strategies [1]. In evaluating the outcomes of these ambulatory surgeries today, the focus is on complications, readmission rates and procedural efficiencies [2]. In contrast, the use of patient-centred parameters like pre-operative anxiety and post- operative satisfaction in evaluating recovery are not being utilised as frequently or as consistently as some of the other measures [3]. Pre-operative anxiety is a common problem found within ambulatory surgical populations. Studies show that there is a relation between pre-operative anxiety and the amount of post-operative pain, the time taken to mobilise after surgery and the time it takes to be ready for discharge after surgery [4]. Recent studies have shown that perioperative education and the provision of psychological preparation are effective at reducing pre-operative anxiety; however, the relationship between assesses pre-operative anxiety scores and the objective post-operative recovery rates still remain undeveloped [4, 5]. Similarly, patient satisfaction is becoming an increasingly recognised marker of quality in ambulatory care; however, this measure is often not considered when compared with measures of quantitative recovery rates [6, 7]. This emerging evidence suggests that pre-operative psychological factors may affect the speed of early mobilisation after surgery, the amount of post-operative analgesia required and the rate of unplanned admissions after outpatient procedures [8, 9-10]. Therefore, it is of interest to evaluate patients in the same laparoscopic day-case surgical cohort using standardised anxiety measurements, structured satisfaction assessments and objective recovery measures concurrently.

## Materials and Methods:

The research described an observational design that operated at a tertiary care facility over the course of eight months; recruiting adults aged 18 to 65 who underwent elective minimally invasive laparoscopic procedures including cholecystectomies and appendectomies. A total of 130 patients were recruited into the study through consecutive enrolment at the day surgery unit. Patients with emergency surgery or who had significant psychiatric illnesses, chronic opioid addiction or ASA physical status scores of three or more were excluded from this study. Ethical approval was provided by the institution/appropriate ethics review board and written informed consent was obtained from all participants prior to their enrolment. To assess the level of anxiety prior to surgery, participants completed the validated state-trait anxiety inventory (STAI-S) on the day of surgery. Based upon previously established cut-offs, patients' scores were categorised as low, moderate or high anxiety. Postoperatively, the patients' level of satisfaction at the time of discharge was measured utilising a structured ten-point Likert-based satisfaction questionnaire, with patients' scores indicating their level of satisfaction. Objective measures of recovery were obtained from four measures: (a) time until the first ambulation postoperatively, (b) postoperative pain as measured on the visual analogue scale (VAS) at six and twelve hours and (c) total analgesics taken postoperatively and (d) time readiness for discharge as measured by a standardised post-anaesthesia discharge score system. Demographic characteristics, time taken to perform the procedure and all intra-operative events were recorded. Descriptive statistics for continuous variables used mean ± SD (standard deviation) and for categorical variables were frequencies and percentages. The association between levels of anxiety, satisfaction and recovery outcomes were evaluated using Pearson Correlation Analysis. Multivariate analysis using multiple linear regression models accounted for age, gender; BMI and duration of surgery were used to identify the independent predictors of time to discharge readiness. Statistical significance was determined at p < 0.05.

## Results:

A total of 130 patients were analyzed, with a mean age of 39.6 ± 11.2 years and 58% females. The mean operative duration was 74.3 ± 18.6 minutes. The mean preoperative STAI-S anxiety score was 44.8 ± 9.7, with 32% classified as high anxiety. The mean postoperative satisfaction score was 38.6 ± 6.4 (maximum 50). The mean time to first ambulation was 5.2 ± 1.4 hours, mean discharge readiness time was 9.6 ± 2.1 hours and mean VAS pain score at 6 hours was 4.8 ± 1.3. Higher anxiety scores were positively correlated with higher VAS pain scores (r = 0.41, p < 0.001) and prolonged discharge readiness (r = 0.37, p < 0.01). Satisfaction scores were negatively correlated with discharge readiness time (r = -0.34, p = 0.001). In multivariable regression, anxiety and satisfaction independently predicted discharge readiness after adjustment for confounders. The mean age (39.6 ± 11.2 years), percentage of females (58%) and mean body mass index (BMI) (25.1 ± 3.2 kg/m^2^) as shown in Table 1 (see PDF), along with the operative duration (74.3 ± 18.6 minutes) and 63% of patients with laparoscopic cholecystectomy performed. Table 2 (see PDF) shows the percentage of patients with high anxiety (32%), moderate anxiety (39%) and low anxiety (29%) prior to surgery. In addition, the STAI-S mean score increased across each anxiety category. Table 3 (see PDF) shows the following: VAS pain scores at 6 hours (4.8 ± 1.3) and 12 hours (3.6 ± 1.1); average time to ambulation (5.2 ± 1.4); and average discharge readiness time (9.6 ± 2.1). Table 4 (see PDF) shows the following positive correlation of anxiety with VAS pain at 6 hours (r = 0.41); discharge readiness time (r = 0.37); and ambulation time (r = 0.29), all of which were statistically significant. Table 5 (see PDF) shows the following negative correlation of satisfaction with discharge readiness time (r = -0.34); VAS pain (r = -0.28); and ambulation time (r = -0.22), which indicates shorter recovery time with greater satisfaction. Table 6 (see PDF) shows that anxiety (β = +0.22, p = 0.002); satisfaction (β = -0.29, p = 0.002); and operative duration (β = +0.18, p = 0.04) are independent predictors of discharge readiness. Age and sex were not significant predictors. Table 7 (see PDF) shows that 74% of respondents reported clear preoperative informational counselling; 52% had concerns about the possibility of postoperative pain; 42% had fears regarding anaesthesia; 78% of respondents expressed high confidence in the surgical team; and 85% of respondents indicated willingness to recommend the procedure.

## Discussion:

The purpose of this research article was to identify the relationship between pre-operative anxiety (POA) and post-operative satisfaction (POS) as independent predictors of early recovery from day-care laparoscopic surgery. There is a clear association between higher scores for POA and higher scores for post-operative pain, longer time before ambulation and longer time until discharge was determined to be ready. Conversely, there was a clear association between higher scores for POS and an earlier time to recovery. As evidenced by these findings, psychological factors are objective predictors of perioperative results, as opposed to subjective perceptions. Thus, the incorporation of a psychological assessment component in conjunction with a more comprehensive preparation for surgery may assist in optimizing post-surgical recovery. The relationship between POA and post-operative pain is consistent with the more recent literature in this area, such as studies demonstrating that an individual with elevated levels of POA has enhanced perceptions of pain due to nociceptive stimulation/analgesia during the post-operative period; therefore, the connection between POA and post-operative recovery is evident in the literature [11]. The sympathetic nervous system becomes activated due to POA, which alters the activity of pain modulation receptors and affects the time it takes for a patient to be able to ambulate. In ambulatory surgery, where an expedited discharge is of utmost importance, even a small delay in ambulation or the ability to control post-operative pain can significantly reduce patient throughput efficiency and, therefore, impede the overall patient experience. Therefore, the current study provides empirical evidence to support this correlation within the context of a large, homogeneous population undergoing day-care laparoscopic surgery [12, 13]. The inverse relationship between POS and time to discharge readiness supports the notion that as patients increase their level of POS, they also reduce their time to discharge and post- operative pain scores. POS likely represents a combination of factors such as how well the patient perceives the quality of communication, whether or not there is congruence between the patient's expectations and the actual level of care received and how confident the patient is in the ability of the surgical team to provide optimal care [14]. As discussed in more detail later in this report, evidence supports the conclusion that providing patients with effective pre-operative education, managing their expectations and providing other forms of support decreases their levels of POA and enhances their recovery process [4, 5-6]. The high percentage of patients who report being confident in their surgical teams and would recommend the procedure also provides additional evidence for the importance of satisfaction based on effective communication [15]. The results of this study confirmed through statistical analysis that both POA and POS were independent predictors of discharge readiness, even when adjusting for demographic variables and length of the surgical procedure. Although the length of the procedure had a minor impact, neither age nor gender was a predictor in this analysis [16]. This supports the hypothesis that the psychological factors may have a more substantial impact on early recovery than specific surgical characteristics. Few studies have examined both standardized measurements of POA, structured measurement of POS and objective measures of time to ambulation and discharge in the same day-care laparoscopic surgical population [17]. In summary, these findings continue to fill the gap in our understanding of how POA and POS correlate with measurable post-surgical recovery endpoints [18]. Although current best practices for providing optimal recovery focus on enhancing analgesic control, promoting early ambulation and optimizing the use of anesthetics, structured psychological education and preparation is not routinely integrated into these preparation protocols for ambulatory surgeries [19]. By demonstrating that POA independently prolongs an individual's time to discharge readiness, this research provides support for implementing pre-operative education, relaxation techniques and managing patients' expectations into the preparation for ambulatory surgical procedures [20]. Finally, the authors acknowledge several limitations of this study, including the limited generalizability of the results due to the single-centre design. The record of POA and POS are based on the patients' own self-reports and could be subject to the influences of their personalities and cultural differences. Additionally, the long-term outcomes of these patients beyond their early discharge were not evaluated. For these reasons, future randomised clinical trials are necessary to determine the effectiveness of targeted interventions to assist patients in reducing their levels of anxiety and implementing structured communication pathways to promote a more economically efficient utilisation of ambulatory surgical facilities. In conclusion, the results of this study support the concept of a clinically relevant biopsychosocial model of the recovery period of ambulatory surgical patients. By addressing patients' psychological preparedness and satisfaction and incorporating them with evidence-based practices in delivering technical excellence, health care professionals may be able to improve and optimise patients' early recovery experiences following surgical procedures.

## Conclusion:

Preoperative anxiety and postoperative satisfaction are independent predictors of early recovery following day-care laparoscopic surgery. Higher anxiety prolongs discharge readiness and increases postoperative pain, whereas greater satisfaction is associated with faster recovery. Integrating structured psychological assessment and counselling into ambulatory surgical pathways may enhance recovery efficiency and patient-centred outcomes.
